# Association of Polymorphisms and Haplotypes in the Insulin-Like Growth Factor 1 Receptor (IGF1R) Gene with the Risk of Breast Cancer in Korean Women

**DOI:** 10.1371/journal.pone.0084532

**Published:** 2014-01-02

**Authors:** Han-Sung Kang, Sei Hyun Ahn, Siddhartha Kumar Mishra, Kyeong-Man Hong, Eun Sook Lee, Kyung Hwan Shin, Jungsil Ro, Keun Seok Lee, Mi Kyung Kim

**Affiliations:** 1 Center for Breast Cancer, National Cancer Center, Goyang-si, Gyeonggi-do, Republic of Korea; 2 Department of Surgery, Division of Breast and Endocrine Surgery, Asan Medical Center, Seoul, Republic of Korea; 3 Division of Cancer Epidemiology, National Cancer Center, Goyang-si, Gyeonggi-do, Republic of Korea; 4 Division of Cancer Biology, National Cancer Center, Goyang-si, Gyeonggi-do, Republic of Korea; MOE Key Laboratory of Environment and Health, School of Public Health, Tongji Medical College, Huazhong University of Science and Technology, China

## Abstract

The insulin-like growth factor (IGF) signaling pathway plays an important role in cancer biology. The IGF 1 receptor (IGF1R) overexpression has been associated with a number of hematological neoplasias and solid tumors including breast cancer. However, molecular mechanism involving IGF1R in carcinogenic developments is clearly not known. We investigated the genetic variations across the IGF1R polymorphism and the risk of breast cancer risk in Korean women. A total of 1418 individuals comprising 1026 breast cancer cases and 392 age-matched controls of Korean were included for the analysis. Genomic DNA was extracted from whole blood and single nucleotide polymorphisms (SNPs) were analyzed on the GoldenGate Assay system by Illumina’s Custom Genetic Analysis service. SNPs were selected for linkage disequilibrium (LD) analysis by Haploview. We genotyped total 51 SNPs in the IGF1R gene and examined for association with breast cancer. All the SNPs investigated were in Hardy-Weinberg equilibrium. These SNPs tested were significantly associated with breast cancer risk, after correction for multiple comparisons by adjusting for age at diagnosis, BMI, age at menarche, and age at first parturition. Among 51 IGF1R SNPs, five intron located SNPs (rs8032477, rs7175052, rs12439557, rs11635251 and rs12916884) with homozygous genotype (variant genotype) were associated with decreased risk of breast cancer. Fisher’s combined *p-*value for the five SNPs was 0.00032. Three intron located SNPs with heterozygous genotypes also had decreased risk of breast cancer. Seven of the 51 IGF1R SNPs were in LD and in one haplotype block, and were likely to be associated with breast cancer risk. Overall, this case-control study demonstrates statistically significant associations between breast cancer risk and polymorphisms in IGF1R gene.

## Introduction

Breast cancer is commonest among malignancies affecting women worldwide and has become second most common cancer among Korean women [1]. Environment, genetics, and immunological defects are major factors in the etiology of breast cancer [2]. Dysregulation of certain growth factors pathways are commonly associated with cancer developments. The insulin-like growth factor (IGF) axis is one of the fundamental cellular pathways regulating proliferation, differentiation, migration, cell survival/apoptosis and transformation. IGF signaling is believed to play a crucial role in cancer and several mechanisms exist by which the IGF signaling is proposed to be dysregulated in breast cancer [3], [4]. The IGF axis includes two IGF ligands (IGF-I and IGF-II), the type one IGF receptor (IGF1R) which mediates the IGF signal, and six IGF binding proteins (IGFBPs) which modulate IGF activities [4], [5]. IGF1R and its ligands insulin-like growth factors (IGF) 1 and 2 serve crucial physiologic roles in growth and development [6], [7]. The IGF pathway also has important pathophysiologic roles in cancer including, neoplastic transformation, higher expression in a variety of neoplasms, and promoting proliferation of neoplastic cells [4], [8].

IGF1 and IGF1R have been reported to play roles in the early transformation of mammary cells, induction of mammary epithelial hyperplasia in a transgenic mouse model, and breast cancer cell growth cells [9]. Overexpression and activation of IGF1R and elevated IGF ligand levels have been observed in a number of human cancers, and aberrant signaling of the IGF system has also been associated with cancer resistance [9], . It was first reported in 1994 that chromosome 15q26, where the insulin-like growth factor 1 receptor (IGF1R) is located, was amplified in <10% of breast cancers [12]. IGF1R amplification was reported at low levels in pancreatic adenocarcinoma xenografts and in two gastric cancer cell lines and in a small percentage of Wilms’ tumors [13], [14]. IGF1R was found overexpressed in gastrointestinal stromal tumors and its aberrant expression might be associated with oncogenesis [15]. The expression patterns of IGF1R in epithelial cells of normal terminal duct lobular units in benign breast biopsies were found to be associated with an increased risk of subsequent breast cancer [16]. In breast cancer, it is evident that IGF1R is involved in resistance to endocrine therapy, anti-human epidermal growth factor receptor 2 (Her2) therapy and chemotherapy [17], [18]. Recently, IGF1R expression was associated with reduced disease-free survival correlating with postoperative recurrence and significantly related to Ki-67 expression in Non-Small-Cell Lung Cancer (NSCLC) [19].

Population-based studies show that circulating IGF-I and IGFBP-3 concentrations as well as genes for IGF and IGF1R have been associated with longevity, cancer and common chronic diseases [10], [20]. Three genome-wide association studies (GWAS) identified seven novel risk loci for breast cancer [21], [22], [23]. A recent study reported the four genome-wide significant loci of the population variation in IGFBP-3 concentration and observed significant association between IGF-I concentration and a locus of FOXO3 associated with longevity [24]. To date, several common genetic susceptibility variants have been identified [25], [26], [27], however, genetic polymorphism in IGF1R are few and remains to be established. IGF1R overexpression is been associated with a number of hematological neoplasias and solid tumors including breast cancer yet been implemented yet the molecular mechanism by which it contributes to carcinogenic developments is clearly not known as well as no universal scoring system has yet been implemented. Also no biomarkers are available yet to select patients who have the potential to benefit from IGF1R-targeted therapy. The comprehensive information from the human genome project and further analyses of GWAS have highlighted single nucleotide polymorphisms (SNP) as the key variations leading to genetic differences in breast cancer susceptibility between individuals. However, the relationship between the IGF1R component of IGF and tumor growth in breast cancer requires more elucidation. Also several genes and SNPs with significant associations with breast cancer risk have been observed yet associations with SNPs of IGF1R have not been elucidated. Since IGF axis appears to play important roles in determining both tumor growth and progression in breast cancer, we aimed to characterize the genetic variations across the IGF1R polymorphism and the risk of breast cancer risk in Korean women. This study allowed us to evaluate the SNPs and haplotypes in the IGF1R gene associated with the risk of breast cancer.

## Materials and Methods

### Ethics Statement

The ethical approval for this study was granted from the institutional review boards at both Asan Medical Center and at National Cancer Center, Republic of Korea. All participants completed in-person interviews with structured questionnaires after they had given informed consent. Participants provide their written informed consent to participate in this study.

### Study Population

A total of 1418 individuals comprising 1026 breast cancer cases and 392 healthy controls of Korean were included for the analysis. The breast cancer cases were recruited from the Breast Cancer Clinic at Asan Medical Center and National Cancer Center, Korea between March 2001 and June 2008. This case population had been identified as having a histopathologically confirmed diagnosis with a first primary breast cancer. Ineligibility criteria included previous malignancy (at either the same site or a different site) and old age greater than 80 years. Control subjects were randomly selected from a cohort of 6,000 health examinees. Controls were women free of any malignancy neoplasms. Data were collected on many potential risk factors of breast cancer, such as years of education, religion, age at menarche, menopausal status, age of first pregnancy, parity, history of benign breast disease, family history of breast cancer, oral contraceptive use, estrogen replacement therapy, smoking, and alcohol consumption.

### Genomic DNA Extraction

Genomic DNA was extracted from 4 ml whole blood using the QIAamp DNA Blood Maxi Kit (Qiagen, Hilden, Germany) according to the manufacturer’s instructions.

### SNP Selection and Genotyping

The selection of specific single nucleotide polymorphisms (SNPs) for this study involved several steps. The list of candidate SNPs was created based on the following criteria: inclusion in the International HapMap Project database [http://www.hapmap.org] with HapMap option for selection of tagging SNP; location within 10 kilobases (kb) of one of the candidate genes (to capture potential regulatory regions) as well as all of the exons and introns; and minor allele frequency of at least 5% in a Han Chinese or Japanese population (to ensure sufficient power). Also included were several non-synonymous SNPs listed on Cancer panel [http://www.illumina.com] on the GoldenGate Assay system by Illumina’s Custom Genetic Analysis service (Illumina Inc., San Diego, CA) that were not in HapMap but had been validated and had a minor allele frequency of above 5% in a Han Chinese or Japanese population. Subsequently, we selected genes that encode proteins in cellular pathways that are likely to be involved in breast carcinogenesis. The major pathways we studied were steroid hormone metabolism and signaling, cytokine and growth factors, transcription factor, double strand break DNA repair, oxidative damage repair, drug response, xenobiotics metabolism, and cell-cycle control. Based on a subsequent screen using PubMed [http://www.ncbi.nlm.nih.gov/pubmed], 285 SNPs were selected from are functional or probably functional, have demonstrated a previous association with breast cancer.

From this list, tagging SNPs were selected using the Tagger program in Haploview (v.3.32). Tagger is a computer program that is used to select and evaluate tagging SNPs based on the empirical patterns of linkage disequilibrium (LD) called ‘bins’. This allows common variation across the region of interest to be captured with fewer SNPs. For this analysis, we used pair-wise tagging to choose SNPs that were correlated at *r* ^2^ equal to 0.98 or greater with all other SNPs in a LD bin. Furthermore, we required that SNPs previously shown to be associated with cancer and nonsynonymous SNPs be ‘forced in’ as tagging SNPs. Completion rates were 99.8% for 98.5% of the assays.

### Statistical Analyses

Haplotypes were constructed from genotypes of 51 SNPs of IGF1R gene by using PHASE [http://linkage.rockfeller.edu]. Linkage disequilibrium (LD) was assessed by Haploview [http://www.broad.mit.edu/mpg/haploview/]. Hardy–Weinberg equilibrium (HWE) for each SNP was tested by comparing the observed and expected genotype frequencies of the controls (χ^2^-test). Differences in demographic variables, selected variables, and frequencies of the genotypes between the cases and controls were evaluated by using the t-test for continuous variables and the chi-square test for categorical variables. Associations with breast cancer risk were evaluated by computing odd’s ratios (OR) and corresponding 95% confidence intervals (95% CI) by logistic regression (PROC LOGISTIC, SAS Version 9.2; SAS Institute, Cary, NC). Covariates considered included age at diagnosis, BMI, age at menarche, age at first parturition. Additive models of effect were applied to all SNPs; tests for trend were conducted by coding for the number of variant alleles and reporting the *p-*value from models based on logistic regression analyses. Dominant or recessive effect models and *p-*values were also calculated when suggested to be appropriate for particular SNPs. Associations between haplotypes and breast cancer risk were analyzed with Haploview; additive, dominant, and recessive models of effect were also evaluated. All statistical tests were two tailed, and *p-*values ≤0.05 were interpreted as statistically significant. The *p-*value for each gene was based on Fisher’s combination.

## Results

### Patient Characteristics

A total of 1418 women were included in the current study; enrolled from two hospitals of the study, National Cancer Center, Ilsan and Asan Medical Center, Seoul, Korea (Table 1). The number of women with breast cancer was 1026 and age-matched control subjects were 392. As expected, breast cancer cases were found to differ from controls in regard to known breast cancer risk factors; cases were having slightly earlier age of menarche, older age at first live birth, a history of breast fibroadenomas, a higher body mass index (BMI) and/or waist-to-hip ratio (WHR) than controls. Clinical and pathological features of breast cancer are also shown in Table 1. Four hundred and fifty three patients (44.5%) had early stage (0+I) carcinoma, 416 patients (40.9%) were diagnosed with disease of stages IIa and IIb, and 149 patients (14.6%) were categorized with IIIa+b+c+IV (III-IV) stages of carcinoma. The state of ER, PR and HER for cases was included in Table 1.

**Table 1:** Characteristics of the subjects in the study and distribution of risk factors.

Characteristics	Control (N = 392)	Case (N = 1026)	*p*-value^1^
*Continuous variable*			
Age at diagnosis, mean (SD), y	46.0(0.5)	46.8(0.3)	0.155
Age at menarche, mean (SD), y	14.6(0.1)	14.4(0.1)	0.039
Age at first parturition, mean (SD), y	25.4(0.2)	26.3(0.1)	<.0001
BMI, mean (SD), kg/m^2^	22.6(0.2)	23.1(0.1)	0.008
<18.5	22(5.6)	36(3.5)	
18.5–22.9	218(55.6)	528(51.4)	
23–24.9	76(19.3)	213(20.7)	
≥25	76(19.3)	249(24.2)	
*Categorical variable*			
Menopausal status, no (%)			
Premenopausal	211(60.1)	706(70.0)	
Postmenopausal	140(39.9)	302(30.0)	0.0006
Oral contraceptive, no (%)			
no	333(86.1)	918(92.6)	
yes	54(14.0)	73(7.4)	0.0001
Hormone replacement therapy status, N(%)			
no	342(89.5)	915(92.6)	
yes	40(10.5)	73(7.4)	0.063
Breastfeeding status, N(%)			
no	106(27.6)	289(29.4)	
yes	278(72.4)	694(70.6)	0.51
Tumor size, cm, no (%)			
≤2		614(59.9)	
>2		411(40.1)	
Node status, no (%)			
N0		635(62.3)	
N1+N2+N3		384(37.7)	
ER positive, no (%)		553(53.9)	
PR positive, no (%)		531(51.8)	
HER2 overexpressing, no (%)		124(24.0)	
Stage, no (%)			
stage 0+I		453(44.5)	
stage IIa+b		416(40.9)	
stage IIIa+b+c+IV		149(14.6)	

*p*-value^1^: Control vs Case. *t* test for continuous variable and χ^2^ for categorical variable.

### IGF1R Genotypes and Breast Cancer Risk

Total fifty one SNPs in the IGF1R gene were examined for association with breast cancer in the study. Information and estimates of frequency and distribution of the 51 IGF1R polymorphisms are shown in Table 2. All the SNPs investigated in the study were in Hardy-Weinberg equilibrium among the control subjects, but four were found to have minor allele frequencies (MAFs) of less than 5% (rs8038056, rs12916884, rs7173377, and rs7166565). As the etiology of breast cancer may differ by menopausal status, stratified analysis was conducted among pre- and post-menopausal status of subjects. However, we observed no statistical difference after adjusting for multiple comparisons in the SNPs and association with the risk of breast cancer among either pre- or post-menopausal women (data not shown). Therefore for further analyses, we choose overall subjects with mixed menopausal status.

**Table 2:** IGF1R allele frequencies and genotype distribution in breast cancer controls and cases.

SNP	Region	Effective	Genotype, (N)Frequency*			HWE *p*
		Allele	Major	Hetero	Minor	
rs4966007	intron	A/G	523(0.37)	657(0.46)	237(0.17)	0.338
rs8028620	intron	T/C	389(0.27)	683(0.48)	344(0.25)	0.286
rs4966009	intron	T/C	529(0.38)	644(0.46)	227(0.16)	0.489
rs8027457	intron	T/C	390(0.27)	683(0.48)	344(0.25)	0.224
rs4966012	intron	C/G	696(0.49)	606(0.43)	115(0.08)	0.414
rs1574213	intron	A/G	885(0.62)	473(0.33)	60(0.05)	0.122
rs11630479	intron	A/G	707(0.50)	595(0.42)	112(0.08)	0.468
rs4966013	intron	A/G	408(0.29)	688(0.48)	320(0.23)	0.733
rs4966015	intron	T/G	844(0.59)	506(0.36)	67(0.05)	0.237
rs8032477	intron	T/C	389(0.27)	729(0.51)	296(0.22)	0.804
rs11634241	intron	A/G	667(0.47)	604(0.42)	147(0.11)	0.570
rs932071	intron	A/G	879(0.62)	473(0.33)	66(0.05)	0.850
rs875686	intron	A/T	818(0.58)	516(0.36)	82(0.06)	0.787
rs1567811	intron	C/G	530(0.37)	696(0.49)	192(0.14)	0.850
rs11632952	intron	A/G	863(0.61)	483(0.34)	72(0.05)	0.868
rs4966024	intron	A/G	364(0.26)	734(0.52)	317(0.22)	0.416
rs8041224	intron	T/C	521(0.37)	671(0.48)	210(0.15)	0.692
rs7175052	intron	A/C	548(0.39)	666(0.47)	203(0.14)	0.345
rs2137680	intron	T/C	379(0.27)	717(0.51)	320(0.22)	0.481
rs12439557	intron	T/C	769(0.54)	538(0.38)	110(0.08)	0.158
rs4966028	intron	A/G	1039(0.73)	336(0.24)	43(0.03)	0.570
rs907806	intron	A/G	1038(0.73)	336(0.24)	37(0.03)	0.622
rs8041953	intron	A/G	1014(0.72)	363(0.26)	39(0.02)	0.760
rs2670501	intron	A/G	529(0.37)	663(0.47)	226(0.16)	0.597
rs4246340	intron	A/C	406(0.29)	707(0.50)	302(0.21)	0.295
rs2684777	intron	T/C	503(0.35)	687(0.48)	228(0.17)	0.187
rs8030950	intron	A/C	828(0.58)	512(0.36)	780(0.06)	0.415
rs12594847	intron	T/C	557(0.40)	646(0.46)	200(0.14)	0.738
rs2684781	intron	T/C	562(0.40)	644(0.45)	211(0.15)	0.451
rs1521481	intron	A/C	810(0.57)	525(0.37)	82(0.16)	0.314
rs4966035	intron	A/G	430(0.30)	663(0.47)	325(0.23)	0.565
rs4966036	intron	T/C	543(0.38)	663(0.47)	211(0.15)	0.480
rs7165875	intron	C/G	419(0.30)	657(0.46)	340(0.24)	0.799
rs3743259	intron	A/G	409(0.29)	667(0.47)	342(0.24)	0.292
rs3743260	intron	A/G	1034(0.73)	345(0.24)	38(0.03)	0.192
rs2684810	intron	T/C	721(0.51)	585(0.41)	111(0.08)	0.454
rs4966039	intron	A/G	950(0.67)	422(0.30)	43(0.03)	0.942
rs2684805	intron	A/G	444(0.31)	701(0.49)	273(0.20)	0.816
rs2684803	intron	A/G	598(0.42)	646(0.46)	171(0.12)	0.233
rs1546713	intron	T/C	596(0.42)	650(0.46)	170(0.12)	0.265
rs7166558	intron	A/G	586(0.41)	659(0.46)	173(0.13)	0.338
rs2229765	coding	A/G	596(0.42)	645(0.46)	171(0.12)	0.285
rs11635251	intron	A/G	659(0.46)	633(0.44)	126(0.10)	0.667
rs8038056	intron	A/G	689(0.48)	604(0.42)	125(0.10)	0.034
rs12916884	intron	T/G	662(0.47)	619(0.44)	136(0.09)	0.047
rs7173377	intron	A/C	658(0.46)	601(0.42)	156(0.12)	0.029
rs7166565	intron	A/G	629(0.44)	622(0.44)	165(0.12)	0.050
rs939626	intron	T/C	819(0.58)	513(0.36)	85(0.16)	0.065
rs12437963	intron	A/G	540(0.38)	668(0.47)	210(0.15)	0.811
rs2684788	flanking_3UTR	A/G	300(0.21)	691(0.49)	424(.030)	0.931
rs1815009	flanking_3UTR	A/G	432(0.30)	686(0.48)	300(0.22)	0.268

Frequency of alleles among total genotyped subjects.

Polymorphisms of interest were then selected for further analysis to address whether associations with breast cancer risk were consistent when stratified by study confounders. Table 3 shows the genotype frequency of each polymorphism in the cases and controls, along with the corresponding ORs after adjusting for age at diagnosis, BMI, age at menarche, and age at first parturition. The additive model of genetics was utilized to calculate the ORs. Of the fifty one SNPs examined in the IGF1R gene, seven intron SNPs (rs8032477, rs4966035, rs2684803, rs1546713, rs7166558, rs11635251, and rs12916884) and one coding SNP (rs2229765) was shown to have a significant association with breast cancer in multivariate analysis. Compared to those with the wild genotype allele, four IGF1R SNPs (rs8032477, rs12439557, rs11635251, and rs12916884) with homozygous genotype (variant genotype) had decreased risk of breast cancer [OR (95% CI): 0.68(0.47–0.98), 0.57(0.36–0.91), 0.57(0.38–0.89), and 0.54(0.35–0.81)]. The multivariate logistic regression analysis by adjusting for age at diagnosis, BMI, age at menarche, and age at first parturition showed evident association of risk with rs11635251 [OR (95% CI): 0.57(0.38–0.89) and rs12916884 (0.54(0.35–0.81)] (Table 3). Intron located eight SNPs (rs907806, rs4966036, rs7165875, rs3743259, rs2684803, rs1546713, rs7166558, and rs2229765) with heterozygous genotypes also had increased risk of breast cancer [OR (95% CI): 0.88(0.65–1.19), 0.92(0.70–1.22), 0.72(0.53–0.97), 0.68(0.50–0.92), 1.35(1.03–1.78), 1.34(1.02–1.77), 1.36(1.04–1.79), and 1.35(1.02–1.77)] (Table 3).

**Table 3:** Analysis of the association between IGF1R gene under additive model and the risk of breast cancer.

	Control			Case						*p-*trend^2^
rs #	Major	Hetero	Minor	Major	Hetero	Minor	Major	Hetero	Minor	
	%			%			OR (CI 95%)^1^			
rs4966007	39.0	45.4	15.6	36.1	46.7	17.2	1 (ref)	1.29(0.98–1.71)	1.41(0.96–2.08)	0.04
rs8028620	28.8	47.2	24.0	27.0	48.6	24.4	1 (ref)	1.27(0.94–1.72)	1.28(0.90–1.84)	0.15
rs4966009	39.9	45.2	14.9	37.0	46.2	16.7	1 (ref)	1.3(0.98–1.72)	1.43(0.96–2.12)	0.04
rs8027457	29.2	46.8	24.0	26.9	48.7	24.4	1 (ref)	1.31(0.97–1.78)	1.27(0.89–1.82)	0.16
rs4966012	47.5	44.1	8.4	49.7	42.3	8.0	1 (ref)	0.89(0.68–1.16)	0.89(0.55–1.43)	0.42
rs1574213	62.5	34.7	2.8	62.3	32.9	4.8	1 (ref)	0.93(0.70–1.22)	1.58(0.77–3.25)	0.75
rs11630479	48.3	43.5	8.2	50.6	41.6	7.8	1 (ref)	0.93(0.71–1.21)	0.87(0.54–1.41)	0.49
rs4966013	29.4	48.9	21.7	28.6	48.5	22.9	1 (ref)	1.10(0.82–1.49)	1.06(0.74–1.52)	0.70
rs4966015	57.8	37.9	4.4	60.2	34.9	4.9	1 (ref)	0.92(0.71–1.21)	1.10(0.59–2.02)	0.82
rs8032477	26.1	49.4	24.6	28.1	52.4	19.6	1 (ref)	1.03(0.76–1.40)	0.68(0.47–0.98)	0.04
rs11634241	44.9	45.2	10.0	47.8	41.7	10.5	1 (ref)	0.92(0.70–1.21)	0.90(0.58–1.40)	0.51
rs932071	59.2	35.7	5.1	63.0	32.5	4.5	1 (ref)	0.90(0.68–1.18)	0.66(0.36–1.18)	0.16
rs875686	54.9	38.8	6.4	58.9	35.6	5.6	1 (ref)	0.91(0.70–1.19)	0.76(0.44–1.32)	0.28
rs1567811	36.5	48.2	15.3	37.8	49.5	12.8	1 (ref)	1.04(0.78–1.37)	0.78(0.52–1.14)	0.34
rs11632952	58.4	36.2	5.4	61.8	33.3	5.0	1 (ref)	0.93(0.71–1.23)	0.64(0.36–1.13)	0.20
rs4966024	24.7	52.0	23.2	26.1	51.9	22.0	1 (ref)	1.02(0.75–1.39)	0.87(0.61–1.26)	0.49
rs8041224	35.0	47.5	17.5	38.0	48.0	14.1	1 (ref)	0.98(0.73–1.30)	0.68(0.46–1.00)	0.08
rs7175052	36.5	45.9	17.6	39.6	47.4	13.1	1 (ref)	1.02(0.77–1.35)	0.61(0.42–0.90)	0.05
rs2137680	26.5	48.2	25.3	26.9	51.5	21.6	1 (ref)	1.16(0.85–1.58)	0.81(0.56–1.15)	0.27
rs12439557	52.3	38.0	9.7	55.0	38.0	7.0	1 (ref)	1.07(0.81–1.40)	0.57(0.36–0.91)	0.18
rs4966028	73.0	24.5	2.6	73.5	23.3	3.2	1 (ref)	0.93(0.69–1.25)	1.43(0.65–3.18)	0.87
rs907806	72.8	25.5	1.8	74.0	23.1	2.9	1 (ref)	0.88(0.65–1.19)	2.07(0.78–5.44)	0.85
rs8041953	70.7	27.0	2.3	72.0	25.0	2.9	1 (ref)	0.92(0.69–1.24)	1.58(0.68–3.67)	0.85
rs2670501	33.9	49.7	16.3	38.5	45.7	15.8	1 (ref)	0.82(0.62–1.08)	0.79(0.54–1.16)	0.15
rs4246340	29.9	51.9	18.2	28.3	49.2	22.6	1 (ref)	1.02(0.76–1.37)	1.27(0.88–1.83)	0.23
rs2684777	33.4	51.5	15.1	36.3	47.2	16.5	1 (ref)	0.90(0.67–1.18)	1.05(0.71–1.56)	0.97
rs8030950	59.7	34.2	6.1	57.9	36.9	5.3	1 (ref)	1.11(0.84–1.46)	0.87(0.51–1.50)	0.87
rs12594847	40.3	45.7	14.0	39.5	46.1	14.4	1 (ref)	1.04(0.79–1.38)	1.02(0.69–1.52)	0.85
rs2684781	39.8	45.2	15.1	39.7	45.5	14.8	1 (ref)	1.03(0.78–1.36)	0.95(0.65–1.39)	0.89
rs1521481	58.1	35.0	6.9	56.8	37.9	5.4	1 (ref)	1.06(0.81–1.40)	0.75(0.44–1.27)	0.69
rs4966035	27.3	51.3	21.4	31.4	45.1	23.5	1 (ref)	0.70(0.52–0.95)	0.90(0.62–1.30)	0.43
rs4966036	36.5	49.2	14.3	39.1	45.8	15.1	1 (ref)	0.92(0.70–1.22)	1.09(0.73–1.62)	0.89
rs7165875	27.3	50.5	22.2	30.4	44.9	24.7	1 (ref)	0.72(0.53–0.97)	0.93(0.64–1.33)	0.55
rs3743259	26.0	52.6	21.4	29.9	45.0	25.5	1 (ref)	0.68(0.50–0.92)	0.96(0.66–1.40)	0.69
rs3743260	74.5	22.7	2.8	72.5	24.9	2.6	1 (ref)	1.11(0.82–1.50)	0.95(0.44–2.03)	0.66
rs2684810	53.1	38.5	8.4	50.0	42.4	7.6	1 (ref)	1.07(0.82–1.40)	0.88(0.54–1.41)	0.93
rs4966039	66.1	30.4	3.6	67.5	29.7	2.8	1 (ref)	0.88(0.67–1.16)	0.75(0.37–1.50)	0.25
rs2684805	33.7	48.2	18.1	30.4	50.0	16.9	1 (ref)	1.26(0.94–1.68)	1.28(0.88–1.86)	0.14
rs2684803	46.2	41.6	12.2	40.8	47.2	12.0	1 (ref)	1.35(1.03–1.78)	1.12(0.75–1.68)	0.18
rs1546713	45.9	41.8	12.2	40.7	47.4	11.9	1 (ref)	1.34(1.02–1.77)	1.12(0.75–1.68)	0.19
rs7166558	45.4	42.4	12.2	39.8	48.0	12.2	1 (ref)	1.36(1.04–1.79)	1.15(0.77–1.73)	0.15
rs2229765	46.2	41.8	12.1	40.7	47.1	12.1	1 (ref)	1.35(1.02–1.77)	1.16(0.77–1.74)	0.15
rs11635251	45.2	43.4	11.5	47.0	45.1	7.9	1 (ref)	0.96(0.73–1.25)	0.57(0.38–0.89)	0.05
rs8038056	50.3	38.0	11.7	48.0	44.3	7.7	1 (ref)	1.28(0.97–1.68)	0.74(0.48–1.17)	0.94
rs12916884	45.4	40.6	14.0	47.2	44.9	7.9	1 (ref)	1.01(0.77–1.33)	0.54(0.35–0.81)	0.03
rs7173377	49.4	38.4	12.3	45.4	44.1	10.6	1 (ref)	1.27(0.96–1.67)	0.95(0.62–1.46)	0.50
rs7166565	47.7	39.5	12.8	43.2	45.6	11.2	1 (ref)	1.29(0.98–1.69)	1.01(0.66–1.53)	0.38
rs939626	56.9	34.7	8.4	58.2	36.7	5.1	1 (ref)	1.02(0.77–1.34)	0.62(0.37–1.05)	0.29
rs12437963	40.3	45.9	13.8	37.3	47.5	15.2	1 (ref)	1.15(0.87–1.52)	1.20(0.81–1.77)	0.28
rs2684788	29.9	49.4	20.7	30.0	48.6	21.4	1 (ref)	0.99(0.73–1.33)	1.06(0.74–1.53)	0.76
rs1815009	31.9	46.7	21.4	30.0	49.0	21.1	1 (ref)	1.15(0.85–1.55)	1.06(0.74–1.50)	0.69

OR (CI 95%)^1^: Multivariate logistic regression model. Adjusted for age at diagnosis, BMI, age at menarche, and age at first parturition.

^2^ Tests for trend were conducted by coding for the number of variant alleles and reporting the *p*-value from models based on logistic regression analyses.

**Figure 1:**
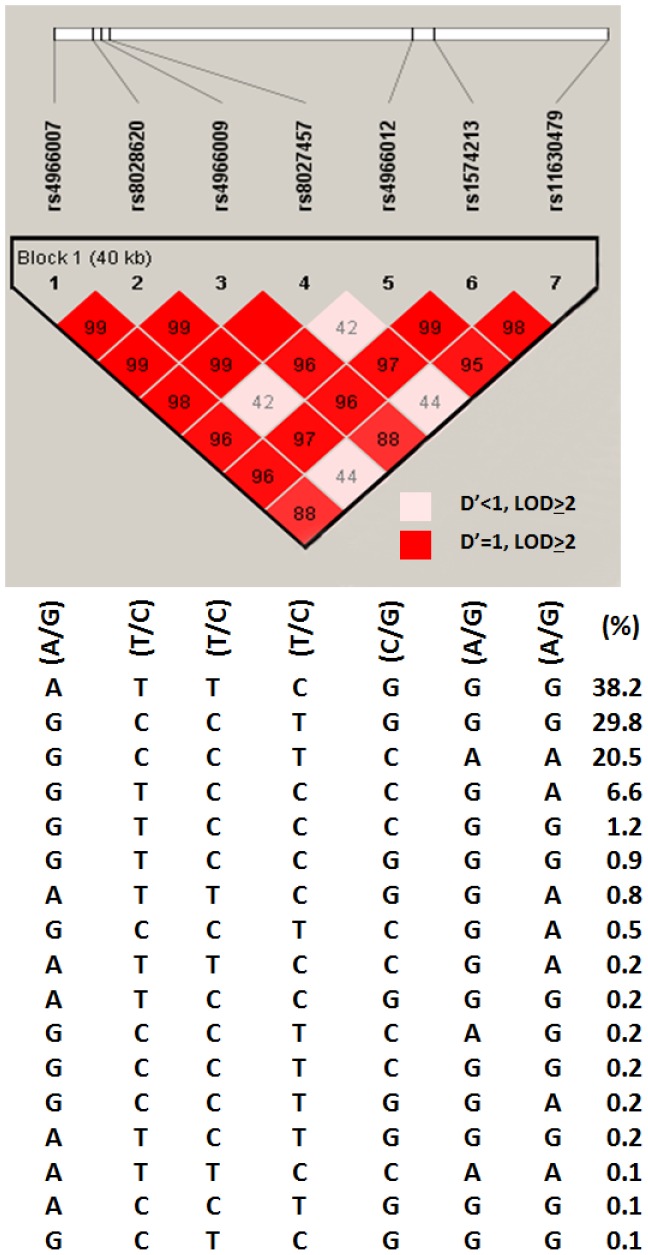
Schematic diagrams of the location of the seven SNPs in IGF1R and strength of the pairwise-linkage disequilibrium (LD) between SNPs. Strengths of the LD between SNPs were indicated by the color scheme, measured using a combination of the statistic D’ and the LOD score.

Next, we evaluated the pairwise linkage disequilibrium (LD) and haplotype distribution among these SNPs. The LD structure of the 7 polymorphic IGF1R SNPs evaluated in the current study is shown in Fig. 1. This LD structure included 1418 genotyped subjects and contained one haplotype block for seven SNPs. The results indicated that seven of the fifty one IGF1R SNPs (rs4966007, rs8028620, rs4966009, rs8027457, rs4966012, rs1574213, and rs11630479) were in LD, and the lowest D’ was greater than 0.90; *r* ^2^ greater than 0.72. Minor allele homozygotes for these SNPs tended to be associated with breast cancer risk and were found to be in one haplotype block (Fig. 1). A significant association with breast cancer risk was found in analysis showing association between IGF1R haplotypes and breast cancer risk. Results from haplotype analysis were generally consistent with results from single SNP analysis; there were significant associations with breast cancer risk. The most common haplotypes in the IGF1R gene were ‘‘AAG’’ and ‘‘GCT’’, which accounted for 84.2% of the cases and 85.5% of the controls (Table 4). The distributions of the other common haplotypes were not significantly different between the cases and controls. Among seven listed SNPs in the haplotype, three SNPs (rs4966012, rs1574213 and rs11630479) provide more risk factor as compared to other four SNPs (rs4966007, rs8028620, rs4966009 and rs8027457).

**Table 4:** Haplotype analysis of the association between IGF1R under three genetic models and the risk of breast cancer.

Block-1	Control		Cases									
	Frequency*		Frequency*	Additive model^a^			Dominant model^b^			Recessive model^c^		
				OR(CI95%)^1^	OR(CI95%)^2^		OR(CI95%)^1^	OR(CI95%)^2^		OR(CI95%)^1^	OR(CI95%)^2^	
rs4966007, rs8028620, rs4966009, rs8027457, rs4966012, rs1574213, rs11630479; intron; 15**												
ACTTCAA	41.1	38.4		1 (ref)	1 (ref)	1 (ref)		1 (ref)	1 (ref)		1 (ref)	
GCCTCAA	44.4	45.8		1.10 (0.86–1.42)	1.31(0.99–1.73)	1.12 (0.88–1.42)		1.33 (1.03–1.73)	1.10 (0.79–1.53)		1.20 (0.83–1.73)	
ATTCGGG	14.5	15.8		1.16 (0.82–1.65)	1.39 (0.94–2.05)							

Percentage of genotype among group.

OR (CI95%)^1^: Univariate logistic regression model.

OR (CI95%)^2^: Multivariate logistic regression model. Adjusted for age at diagnosis, BMI, age at menarche, and age at first parturition.

^a^ Breast cancer risk for heterozygotes (AB) and homozygotes (BB), each compared with major allele homozygotes (AA) in model adjusted genotyping.

^b^ Breast cancer risk for minor allele carriers (AB/BB) compared with major allele homozygotes (AA) in model adjusted for genotyping.

^c^ Breast cancer risk for minor allele homozygotes (BB) compared with major allele carriers (AA/AB) in model adjusted for genotyping.

SNPs’ location on chromosome number.

## Discussion

The IGF pathway and its receptor component IGF1R appears to play important roles in breast cancer progression and tumor growth. A case-control study was conducted to first comprehensively evaluate IGF1R genetic variants in relation to breast cancer risk and then to assess any promising associations among study population. Four IGF1R SNPs (rs8032477, rs12439557, rs11635251, and rs12916884) were found to be associated with breast cancer risk in the study populations as well as significant in combined analyses (*p* = 0.0003). Eight SNPs with heterozygous genotypes also had decreased risk of breast cancer. Considering the status of IGF1R polymorphisms our results may help in improving the ability to develop effective treatment modalities with reference to associated risk breast cancer. To the best of our knowledge, these IGF1R gene polymorphisms have not been previously evaluated for associations with breast cancer susceptibility.

Breast cancer is commonest among malignancies affecting women worldwide. Concurrent increase in incidences has made breast cancer one of the most frequently occurred diseases among Korean women [1]. The IGF1R is a tyrosine kinase growth factor receptor that when activated plays an important role in signal transduction pathways regulating cell proliferation, survival migration, and differentiation [25]. Upon ligand binding, IGF1R stimulates downstream pathways responsible for these processes via phosphorylation of substrates including insulin receptor substrate 1 (IRS1), Shc, and phosphatidyl inositol-3 kinase (PI3K). The differential staining patterns for IGF1R showed various possible mechanisms including one intriguing possibility of differential activation status of IGF1R [16]. In several reports [16], [27], [28], the absence of serum or IGF ligands display a predominantly membranous staining pattern for IGF1R; this localized membrane staining pattern is less prominent following ligand stimulation. Ligand stimulation induces internalization of IGF1R and other receptor tyrosine kinases like Met and EGFR [29], [30]. The activation of specific signaling pathways is considered regulatory mechanism such as internalization of IGF1R after IGF1 stimulation in oligodendrocyte precursor cells was required for phosphorylation and sustained activation of Akt [31]. The role of IGF pathway also exists in prostate carcinogenesis with substantial epidemiologic and experimental evidences showing an increased risk, especially advanced disease among men with high IGF-I blood levels [32], [33], [34]. Recently Fu et al. [35] indicated a correlation between IGF1R expression in primary breast cancer and suggested IGF1R as a good prognostic marker. In addition, IGF1R has emerged as one of the most promising molecular targets in cancer treatment with several technologies being employed in its downregulation [36].

In this study, we conducted a thorough analysis of common genetic variations in breast cancer controls and cases in Korean women. We analyzed the association between IGF1R SNPs and breast cancer risk following multiple testing corrections. Breast cancer is a heterogeneous disease with different tumor subtypes, we focused on the effect of IGF1R in a mixed population of Korean women with mixed status of ER, PR, HER, and triple negative tumor subtypes. Considering the co-effect of IGF1R gene, several risk factors were used in the multivariate analysis. The SNPs of IGF1R in the breast cancer subjects was positively associated with subsequent risk and women with IGF1R SNPs were at higher risk of developing breast cancer. Seven IGF1R SNPs in intron region (rs8032477, rs4966035, rs2684803, rs1546713, rs7166558, rs11635251), and one in coding region (rs12916884) were significantly associated with the breast cancer risk. Also four IGF1R SNPs with homozygous genotype (variant genotype) had decreased risk of breast cancer. The multivariate analysis adjusted for age at diagnosis, BMI, age at menarche, and age at first parturition manifested the association of risk with rs11635251 (0.57(0.38–0.89)) and rs12916884 (0.54(0.35–0.81)). The pairwise LD and haplotype distribution analyses showed that seven polymorphic IGF1R were in LD and in one haplotype block. These seven IGF1R SNPs (rs4966007, rs8028620, rs4966009, rs8027457, rs4966012, rs1574213, and rs11630479) were likely to be associated with breast cancer risk. The most common haplotypes in the IGF1R gene were ‘‘AAG’’ and ‘‘GCT’’ among over all subjects in the study. Overall, these results confirmed a significant association between IGF1R SNPs and the risk of breast cancer.

Cytoplasmic IGF1R expression in epithelial cells of normal breast tissue has been positively associated with subsequent risk of breast cancer [37]. However, Hartog et al. [38] reported that cytoplasmic IGF1R expression in ER-positive invasive ductal breast carcinomas is associated with a more favorable prognosis. However, a recent study did not correlate with IGF1R with any prognostic indicators or outcome [39]. In addition, membrane IGF1R correlated with larger tumor size and with younger patient age at diagnosis, suggesting that IGF1R may be associated with a negative prognosis. Membrane IGF1R expression has recently been associated with poor prognosis in ER-negative invasive breast cancers [38]. However, membrane IGF1R positivity also correlated with lower tumor grade in a recent study [39], which would be indicative of a favorable prognosis. Recent research showed that IGF1R expression was strongly related to a shorter disease-free survival in triple-negative breast tumors [40]. However, in the present study, no prognostic significance was evaluated for the IGF1R so further study may help to explain the associations of IGF1R with breast cancer in individuals with different tumor subtypes. These observations together with our epidemiological findings demonstrate that IGF1R additionally represents a promising target for the development of novel anti-cancer therapeutics. Because IGF1R could also serve as a supplementary target, anti-IGF1R therapy is expected to appear as a supplementary approach in combination with other therapeutic strategies. In addition, these observations may also serve as background information for selecting patients for IGF1R-targeted therapy. Other epidemiologic studies performed at similar levels provide inputs important clinical suggestions in management of breast cancer. Such as haplotypes and polymorphisms in X-ray repair cross-complementing 1 gene may be a genetic determinant for developing breast cancer [41]. Also GWAS identified genetic variants of solute carrier family 4, sodium bicarbonate cotransporter, member 7 gene and their associations with increased risk of breast cancer [42].

A limitation of this study is smaller sample size and thus statistical power of our study was limited in the stratified analyses in the small sample size of the subgroups. Although this study reports genetic polymorphisms on the IGF1R on breast cancer in Korea for the first time, the findings need to be validated in further studies with larger sample size or in meta-analyses, which is also aimed in our laboratory for years to come.

## Conclusions

This case-control study identifies tagSNPs in the IGF1R gene and demonstrates their statistically significant associations with the risk of breast cancer risk in a population of Korean women. The identification of single biomarker correlating with the response to IGF inhibitors is unlikely. However, the status of IGF1R gene polymorphisms and their plausible roles in the IGF signaling promoting cancer growth augments the importance of our findings. This may help in improving the ability to develop effective treatment modalities for those conditions in which IGF1R gene is involved. To the best of our knowledge, this study is the first to demonstrate the association between IGF1R polymorphisms with the risk of breast cancer.
