# Correction: Association of Polymorphisms and Haplotypes in the Insulin-Like Growth Factor 1 Receptor (IGF1R) Gene with the Risk of Breast Cancer in Korean Women

**DOI:** 10.1371/journal.pone.0091218

**Published:** 2014-03-14

**Authors:** The PLOS ONE Staff

In the Funding statement, an additional grant from the Korea National Cancer Center is incorrectly omitted. The Funding statement should read: "This study was supported by grant-in-aids from the Korea National Cancer Center (grant nos. NCC-0910310, NCC-1310361 and NCC-0910220) and a grant (2009-022) from the Asan Institute for Life Sciences, Seoul, Republic of Korea. The funders had no role in study design, data collection and analysis, decision to publish, or preparation of the manuscript."
